# Self–reported diabetes education among Chinese middle–aged and older adults with diabetes

**DOI:** 10.7189/jogh.06.020402

**Published:** 2016-12

**Authors:** Hanzhang Xu, Jianfeng Luo, Bei Wu

**Affiliations:** 1 Duke University School of Nursing, Durham, North Carolina, USA; 2 Department of Health Statistics and Social Medicine, School of Public Health, Fudan University,130 Dongan Road, Shanghai, China; 3 Duke University Center for the Study of Aging and Human Development, Durham, North Carolina, USA; 4 Duke Global Health Institute, 310 Trent Drive, Durham, North Carolina, USA

## Abstract

**Background:**

To compare self–reported diabetes education among Chinese middle–aged and older adults with diabetes in three population groups: urban residents, migrants in urban settings, and rural residents.

**Methods:**

We used data from the 2011 China Health and Retirement Longitudinal Study. The sample included 993 participants age 45 and older who reported having diabetes diagnosed from a health professional. We performed multilevel regressions performed to examine the associations between characteristics and different aspects of diabetes education received.

**Findings:**

Our study shows that 20.24% of the participants received no diabetes education at all. Among those who received information, 46.82% of respondents with diabetes received weight control advice from a health care provider, 90.97% received advice on exercise, 60.37% received diet advice, 35.12% were spoken to smoking control, and only 17.89% of persons were informed of foot care. After controlling socioeconomic factors, life style, number of comorbidities and community factors, we found that compared with migrant population and rural residents, urban residents were more likely to receive diabetes education on diet. Urban residents were also more likely to obtain diabetes education and more aspects of diabetes education comparison with migrants and rural residents.

**Conclusions:**

Our study suggests diabetes education is a serious concern in China, and a significant proportion of the participants did not receive advice on smoking control and foot care. Rural residents and migrants from rural areas received much less diabetes education compared with urban residents. Efforts to improve diabetes educations are urgently needed in China.

The prevalence of diabetes has dramatically increased globally, especially in China [1]. The prevalence of diabetes among the Chinese adult population has significantly increased from 5.5% to 11.6% during the past decade [2,3]; a total of 114 million adults have diabetes. Previous studies have shown that diabetes education, serving as the keystone of diabetes self–management, provides diabetics adequate knowledge and tools to facilitate them monitoring blood glucose value, preventing the complications of diabetes, and eventually improving their quality of life [4-6]. Literature on diabetes education in China focused on the diabetes education receipt rate within people living in a certain region [7-13]. So far, only two previous studies reported the current status of diabetes education at national level [8,13]. These studies showed that over three quarters of the diabetics in China reported having received diabetes education and most of them obtained such education from a health profession. However, both of these studies were limited by a lack of explicit measures of the aspects of diabetes education and its associated factors that impact people receiving diabetes education.

In addition, disparities in health care systems between Chinese urban and rural areas may explain the variation in awareness of diabetes and access to health care, thus affecting people receiving diabetes education [14-16]. To our knowledge, no study has been done to examine diabetes education across place of residence, such as urban and rural settings. Also the urbanization process in China resulted in the dramatic growth of the internal migrants. This migrant population has grown to 221 million in 2010, the majority of which (72%) were from rural areas [17]. Migrants in China are likely to encounter hostility and discrimination from urban residents. For example, many jobs that migrants took are limited to certain types that urban residents are not willing to do. They are often denied access to many of the social and medical programs such as health insurance and unemployment benefits that their urban counterparts are entitled to have [18]. With low socioeconomic status and limited medical insurance, these migrants had limited access to health care, which would have negative impacts on the migrant workers’ health status [18,19]. Previous research that targeted this population mainly focused on communicable diseases such as HIV/AIDS and occupational diseases [20,21]. No published literature has explicitly focused on chronic diseases education such as diabetes among migrant population in China. Considering the sheer size of the migrant population in China and the impact of urbanization on people’s life style, it is pressing to examine the prevalence of non–communicated diseases especially diabetes among the migrant population.

The goal of this study is to investigate the variation of types of diabetes education received among Chinese middle aged and older adults with diabetes across three groups: urban residents, rural residents, and rural–to– urban migrants. We aim to address this knowledge gap by using a national sample with individual– and community–level data.

## METHODS

### Data source

Data were applied from the 2011 China Health and Retirement Longitudinal Study (CHARLS) data set for this study. CHARLS is a biennial study that aims to collect data in three domains–– health, financial, and family––from a nationally representative sample of Chinese residents age 45 and above [22]. The CHARLS National Baseline (2011 wave) was conducted in 28 out of 30 provinces in China and collected both individual– and community–level information from 17 708 individuals living in 10 287 households. The questionnaire included modules like family structure/transfer, health status and functioning, biomarkers, health care and insurance, work, retirement and pension, income and consumption, and assets (individual and household). The overall response rate for the 2011 CHARLS was over 80%: 94% in rural areas compared with 69% in urban areas [22]. In our study, we included 993 respondents who reported having diagnosed diabetes from a health care provider.

### Outcome measures

This study used seven diabetes education variables as the study outcomes: diabetes education received (Yes/No), five aspects of diabetes education: Weight control (Yes/No), Diet (Yes/No), Exercise (Yes/No), Smoking control (Yes/No), and Foot care (Yes/No), and a sum of diabetes education received. In the 2011 CHARLS questionnaires, the presence of received diabetes education was determined by the following question “Has your care providers ever given you diabetes education/advice on the following?” Possible answers included: weight control, diet, exercise, smoking control, foot care, and none of them above. Respondents were classified as having received diabetes education (Yes) if they chose at least one aspect of the five diabetes education. We also operationalized a variable regarding a sum of diabetes education received based on how many aspects of diabetes education each individual chose. Additionally, among those respondents who received at least one aspect of diabetes education, the researchers generated five dummy variables for each aspect.

### Condition specific measures

*Condition specific variables* included age, gender, smoking status (current smoker or not), drinking habit (Yes/No), defined as women have more than one drink per day while men have no more than two drinks per day [23], body mass index BMI (normal, overweight, and obese), categorized according to the BMI cutoff points for Asians, physical activities (Yes/No), years of being diagnosed of diabetes (1 = 0–1 year, 2 = 1–5 years, 3 = 5 years and above), and number of comorbidities (including hypertension, dyslipidemia, cancer or malignant tumor, chronic lung diseases, liver diseases, heart problems, stroke, kidney diseases, stomach or other digestive disease, emotional, nervous, or psychiatric problems, memory–related disease, arthritis or rheumatism, and asthma).

### Physical and social environment

*Physical and social environment* included both individual– and community– level variables. The individual level variable was the place of residence, which was coded into three categories: 1 = urban residents, 2 = migrant population, and 3 = rural residents. *Urban residents* included respondents living in urban areas with urban medical insurance. *Migrant population* was defined as respondents who live in an urban area with rural medical insurance, in most cases the New Cooperative Medical Insurance. *Rural residents* were respondents living in rural areas with rural medical insurance. Two community–level variables were 1) Physical accessibility of health facilities (Yes/No), which was defined by World Health Organization (WHO) that households that live within 15 minutes travel time to any public or private health facility, and 2) Use of community level health facilities (Yes/No). These community–level characteristics were extracted from the CHARLS community survey.

### Individual and family factors

*Individual and family* factors included marital status (married vs other), education level, and household income. Because of the extremely low levels of education among Chinese older adults, education was categorized into four levels as illiterate (No formal education/illiterate), primary education only school (did not finish primary school but capable of reading and/or writing, Sishu/home school, elementary school), secondary education but no higher (middle school, high school, vocational school), and college level and above (two–/three–year college/associate degree, four–year college/Bachelor’s degree, Master’s degree and Doctoral degree/PhD). Household income included the following dimensions: wage income, self–employment income, agricultural income, pension income, and transfer income. In this analysis, the household income was aggregated into three levels: low = 0% to 33.3%, middle = 33.4% to 66.6%, high = 66.7% to 100%.

### Statistical analysis

Descriptive statistics of individual and community factors and were examined by using *t* and χ^2^ test procedures to compare mean differences and frequency distributions. Then, two–step multilevel regression models were applied in the study. First, logistic regression models were used to examine the outcome of having diabetes education and regression models were used to examine the outcome of the sum of diabetes education received. Then, a series of logistic regression models were applied to test the each aspect of diabetes education. STATA 13 (College Park, TX, USA) was used to analyze data with a significance level of 0.05.

## RESULTS

### Bivariate analysis

**Table 1** shows the sample demographic and health related characteristics. The mean age of the sample was 62 years, and 54.55% were women; 19.77% of participants were migrants, 46.78% were urban residents, and 33.45% were rural residents. Overall 20.24% of the participants received no diabetes education at all. Among participants who received at least one aspect of diabetes education, 46.82% of respondents with diabetes received weight control advice from a health care provider, 60.37% received diet advice, 90.97% received advice on exercises, 35.12% were suggested about smoking control, and only 17.89% of persons were informed of foot care.

**Table 1:** Sample characteristics, China Health and Retirement Longitudinal Study (CHARLS), 2011

	Weight control	Diet	Exercise	Smoking control	Foot care	None	Total (n = 993)
**Individual characteristics:**							
Age (mean)	60.76†	61.44	61.84	60.00‡	59.79*	63.82	62.16
Female	44.79*	47.04†	52.65	21.96‡	41.23†	65.41†	54.55
Married	83.7	85.6	85.18	85.4	92.29*	78.97	84.36
**Education:**							
Illiterate	16.27*	17.11‡	21.85	11.06†	13.54	29.32*	23.1
Primary only	32.52	31.33	35.55	35.56	40.47	41.68	36.43
Secondary but no higher	45.37	45.4	37.93	26.25	38.25	24.57	35.76
College and above	5.84	6.16	4.68	7.13	7.74	4.43	4.8
**Household income:**							
Low	33.94	39.12	36.71	31.31	35.54	40.24†	37.77
Middle	27.13	30.75	25.07	26.8	25.34	36.11	27.13
High	38.93	30.13	38.22	41.89	39.12	23.65	35.1
**Place of settings:**							
Migrants	20.1	17.67‡	19.55†	21.52	12.70*	20.46‡	19.77
Urban	52.78	59.27	50.67	47.69	60.35	31.76	46.78
Rural	27.11	23.06	29.78	30.8	26.95	47.79	33.45
**Health status:**							
Current smoker	23.71	20.67	17.74	42.47‡	31.66†	20.18	19.4
Drinking	4.78	4.73	3.87	8.7	2.84	5.11	4.34
Exercise	12.82	12.77	14.47	11.71	16.86	17.7	15.01
**Years of diabetes:**							
0–1	17.33†	37.13‡	24.52*	17.84†	17.28	35.95*	27
1–5	38.4	28.18	33.25	41.02	36.42	25.06	31.46
≥5	44.26	34.69	42.23	41.15	46.3	38.99	41.54
**Body mass index:**							
<24	32.91	41.02	37.74	38.75	32.77	41.03	39.07
24–27.9	40.53	40.77	40.36	37.09	42.25	39.98	39.94
≥28	26.56	18.21	21.89	24.16	24.97	18.99	20.99
Number of comorbidities (mean)	2.32	2.33	2.39	2.39	2.27‡	2.36	2.38
**Community characteristics**							
Access to health facilities	90.01	90.52*	88.92	88.95	88.38	82.85*	88.01
Use of community health facilities	74.13	74.87	73.64	75.5	69.96	74.78	73.6

*P < 0.05.

†*P* < 0.01.

‡*P* < 0.001.

Respondents who reported being informed by a health care provider about diabetes education on diet, exercise, and foot care were more likely to be urban residents. A higher proportion of males, better educated, and with higher income received at least one aspect of diabetes education. Additionally, people who didn’t receive any diabetes education were more likely to be those who had diabetes within a year. In addition, respondents who lived in a community with accessible health facilities were more likely to receive diabetes education.

### Multivariate analysis

**Table 2** presents the results of step 1 regression models. Results show that in comparison with urban residents, both migrant population (adjusted odds ratio (OR) = 0.44, 95% confidence interval (CI) 0.23–0.83) and rural residents (adjusted OR = 0.43, 95% CI 0.24–0.77) were less likely to obtain diabetes education. We also found older respondents were less likely to receive any diabetes education (adjusted OR = 0.96, 95% CI 0.93–0.98). Gender was another significant factor for receiving diabetes education: females tended to receive no diabetes education (adjusted OR = 0.40, 95% CI 0.23–0.67) compared with their male counterparts. Additionally, people with more comorbidities were more likely to receive diabetes education (adjusted OR = 1.15, 95% CI 1.01–0.31).

**Table 2:** Regression on receiving diabetes education and sum of diabetes education, China Health and Retirement Longitudinal Study (CHARLS), 2011

Variables	Receiving diabetes education (AOR, 95% CI)	Sum of diabetes education (β)
**Individual characteristics:**		
Age	0.96 (0.93–0.98)†	–0.17†
Female	0.40 (0.23–0.67)‡	–0.19†
Married	1.11 (0.60–2.04)	0.01
**Education (vs illiterate):**		
Primary only	0.91 (0.55–1.50)	–0.01
Secondary but no higher	1.22 (0.66–2.27)	0.09
College and above	0.55 (0.15–1.95)	0.02
**Household income (vs low):**		
Middle	1.05 (0.49–1.41)	0.03
High	1.11 (0.67–1.68)	–0.00
**Place of settings (vs urban):**		
Migrant	0.44 (0.23–0.83)*	–0.16†
Rural	0.43 (0.24–0.77)†	–0.14*
Current smoker	0.63 (0.36–1.11)	0.07
**Years of diabetes (vs 0–1):**		
1–5	1.41 (0.86–2.31)	0.19‡
≥5	1.16 (0.72–1.88)	0.17†
**Body mass index (vs <24):**		
24–27.9	0.84 (0.54–1.32)	0.06
28 and above	0.89 (0.52–1.52)	0.13*
Drinking	0.59 (0.24–1.46)	–0.09
Exercise	1.01 (0.61–1.65)	–0.06
Number of comorbidities	1.15 (1.01–1.31)*	0.15†
**Community characteristics:**		
Access to health facilities	1.42(0.81–2.49)	0.07

CI – confidence interval, AOR – adjusted odds ratio

*P < 0.001.

†*P* < 0.05.

‡*P* < 0.01.

Similar results were found in the sum of diabetes education received: rural residents (β = –0.14, *P* = 0.04) along with migrants (β = –0.16, *P* = 0.006) from rural area were more likely to receive less diabetes education compared to urban residents. People who were female (β = 0.19, *P* < 0.001) and older (β = –0.17, *P* = 0.002) reported receiving fewer diabetes education contents. People with more comorbidities were more likely to receive more aspects of diabetes education (β = 0.15, *P* = 0.002). Additionally, people with longer period of diagnosis of diabetes: having diabetes for more than one year were more likely to obtain more aspects of diabetes education from a health professional.

**Table 3** summarizes the results of the step 2 logistic regressions. First, both rural residents (adjusted OR = 0.41, 95% CI 0.20–0.82) and the migrant population (adjusted OR = 0.41, 95% CI 0.18–0.90) were less likely to receive diet education. Also rural residents were more likely to receive diabetes education in terms of smoking control (adjusted OR = 2.70, 95% CI 1.13–6.42). Second, for weight control, people who were overweight or obese, or having diabetes for more than five years were more likely to receive this education. For smoking control, higher level of education, male, current smokers, obesity, and having diabetes for more than one year were positively related to receiving diabetes education on smoking control. In addition, we found that respondents who received foot care education were more likely to be current smokers (adjusted OR = 2.70, 95% CI 1.38–5.31) along with people having more comorbidities (adjusted OR = 1.20, 95% CI 1.03–1.40). Although people living in communities with more accessible health facilities tended to have better diabetes education about diet and foot care, the results were not significant across all aspects of diabetes education in the full model.

**Table 3:** Logistic regression models on types of diabetes education received, CHARLS, 2011

Variables	Weight control (AOR, 95% CI)	Diet (AOR, 95% CI)	Exercise (AOR, 95% CI)	Smoking control (AOR, 95% CI)	Foot care (AOR, 95% CI)
**Individual characteristics:**					
Age	0.98 (0.95–1.01)	0.97 (0.94–1.00)*	0.97 (0.93–1.01)	0.97 (0.93–1.01)	1.00 (0.96–1.03)
Female	0.92 (0.54–1.56)	0.96 (0.54–1.71)	0.98 (0.43–2.24)	0.16 (0.08–0.33)†	0.92 (0.49–1.71)
**Education (vs illiterate):**					
Primary only	0.76 (0.40–1.42)	1.18 (0.62–2.24)	2.21 (0.85–5.71)	1.01 (0.42–2.41)	0.85 (0.40–1.81)
Secondary but no higher	1.28 (0.64–2.58)	1.87 (0.90–3.88)	0.90 (0.35–2.33)	1.41 (0.54,3.70)	1.01 (0.44–2.31)
College and above	1.51 (0.38–6.06)	2.66 (0.48–14.84)	2.43 (0.25–23.36)	9.46 (1.50–59.69)*	1.31 (0.28–6.07)
**Place of settings (vs urban):**					
Migrant	0.93 (0.46–1.89)	0.41 (0.18–0.90)*	0.77 (0.31–1.92)	1.62 (0.59–4.45)	0.42 (0.17–1.04)
Rural	1.12 (0.60–2.08)	0.41 (0.20–0.82)*	1.32 (0.57–3.05)	2.70 (1.13–6.42)	0.80 (0.40–1.60)
Current smoker	1.76 (0.94–3.28)	1.25 (0.64–2.44)	0.53 (0.23–1.23)	17.85 (6.89–46.26)‡	2.70 (1.38–5.31)†
**Years of diabetes (vs 0–1):**					
1–5	2.91 (1.60–5.28)†	2.70 (1.48–4.95)†	1.39 (0.62–3.10)	2.21 (1.01–4.85)*	1.9 (0.94–3.82)
≥5	3.40 (1.89–6.11)‡	2.95 (1.63–5.36)‡	1.64 (0.73–3.69)	3.10 (1.39–6.88)†	1.73 (0.87–3.41)
**Body mass index (vs <24):**					
24–27.9	2.10 (1.27–3.49)†	1.31 (0.77–2.23)	1.16 (0.57–2.37)	1.66 (0.83–3.33)	1.02 (0.56–1.84)
≥28	3.99 (2.13–7.49)‡	2.10 (1.09–4.04)*	1.17 (0.50–2.75)	2.33 (1.06–5.15)*	1.55 (0.79–3.04)

CI – confidence interval, AOR – adjusted odds ratio

*P < 0.05.

†*P* < 0.01.

‡*P* < 0.001.

## DISCUSSION

Our study is one of the first to examine the factors affecting people receiving diabetes education in China using a national representative sample. The results showed great variation in the receipt of diabetes education. Additionally, data from this study revealed that place of residence plays an important part in determining whether one will receive diabetes education and the aspects of diabetes education.

National standard guideline for diabetes education from the American Diabetes Association (ADA) and the China Diabetes Society (CDS) suggest that individualized diabetes education may cover core topics such as nutritional management, physical activities, and preventing, detecting, treating complications [23,24]. Our study indicated that diet and exercise advices were the two most common diabetes education topics among all the five. However, smoking control and foot care education were the lowest two, which are more involving lowering the risk factors that damage blood vessels as well as preventing or delaying complications. Smoking is prevalent in China—the world largest tobacco consumer [25]. However, only 35.12% of the Chinese adults were aware of the health hazards related to smoking. Less than 50% of the current smokers in our study reported receiving quit smoking education, especially among urban residents. Thus, diabetes education regarding avoiding tobacco use is urgently needed. Apart from smoking, our results showed that diabetic patients in China received limited education on foot care, which is consistent with previous study [13]. People with diabetes could develop a series of foot problems including nerve damage, skin changes, and foot ulcer [26]. These complications could be easily prevented by performing regular foot care [26]. Guidelines for standard medical care for diabetes from the ADA and CDS [23,24], recommend that health professionals should provide foot care education to all diabetic patients. As a result, our findings call for future comprehensive diabetes education with a particularly focus on foot care.

Our study showed that residential settings are related to receipt of diabetes education. Migrant population and rural residents compared with urban residents were less likely to receive any diabetes education and less aspects of diabetes education. Diabetic patients living in rural areas and migrant population were less likely to be educated about diet in comparison with their urban counterparts. These results were consistent with our exploratory analysis that identified the association between medical insurance type and receipt of diabetes education, which we found that people with urban employee or government medical insurance were more likely to receive diabetes education about diet, exercise, and foot care. It is possible that these disparities by place residence were partially due to medical insurance type. In comparison with New Rural Cooperative Health Insurance, urban employee and government medical insurance have higher outpatient coverage and help people to access to health services [27,28]. Previous studies have shown that individuals with urban employee and government health insurance were more likely to use outpatient services [27,29]. Diabetes education, as part of chronic disease management, would happen during outpatient visits. As a result, diabetic patients with health insurance that has a higher coverage of outpatient visit would have more outpatient care utilization, and subsequently would be more likely to receive diabetes education.

Besides the differences in health insurance, disparities of health resources may also relate to the receipt of diabetes education. Residents in urban area were more likely to see a doctor in tertiary or secondary hospitals, where diabetes education programs or certified diabetes educators were available. While rural residents and migrant population were more likely to use village clinics, township hospitals, or private clinics that lack of professional doctors and nurses, especially trained diabetic health care providers, which add another barrier of receiving diabetes education [27-29].

Aiming at providing universal health coverage, the Chinese government has launched different public health insurance programs for various populations, with a particular focus on the rural population [28]. Despite the rapid expansion of insurance coverage, with over 96% New Cooperative Health Insurance coverage among rural population, Chinese adults from rural areas still have a relatively high out–of–pocket payment during outpatient visits [28]. Regional disparities of health resources and health professional still existed [28]. Given the great epidemic of non–communicable diseases in China, such as diabetes, there is an urgent need to expand outpatient care coverage for rural population as well as improve quality of health services especially in rural community health centers. The government should focus on increasing reimbursement rates for outpatient care, especially for those from rural areas or migrant populations with chronic diseases. Community capacity building should also be prioritized to promoting non communicable disease prevention and control. In addition to the policy and programs mentioned above, providing flexible reimbursement plans that would cover outpatient care both in rural and urban settings for migrant population may also encourage this segment of the population to use outpatient health promotion services, such as diabetes education classes when needed.

Twenty percent of the diabetics did not receive any diabetes education, which is similar to the rates reported in previous studies [8,13]. Participants with no diabetes education were more likely to be in a migrant population or rural residents, older, or females. These findings were consistent with prior research that age and gender are significant factors associated with not receiving diabetes education. Older adult and females have a higher prevalence of diabetes [27], further diabetes education interventions should be more targeted to reach this group of people.

In our study, no significant association was found between community characteristics (access to health care facility, use of community health facility) and receipt of diabetes education. The lack of strong association could be partially contributed to the fact that most community health centers lack of qualified professional health care providers to provide health education related to prevention and control of non–communicable diseases, such as diabetes [28]. Previous studies provided evidence that the majority of health professionals who worked in primary health care facilities only received 2–3 years basic medical training [30,31]. The shortage of professional trained health care providers could limit the community health centers’ capacity in providing adequate education to tackle the issue of non–communicable disease prevention. Further research is needed to assess whether access to different types of health care facilities is associated with the receipt and quality of diabetes education.

Findings in this study support that contextual factors (age, gender, comorbidities, year of diabetes) were associated with individuals receiving diabetes education, which is consistent with prior research [8,13]. We also found that other contextual factors, an individual’s health behaviors, were associated with the aspects of diabetes education that health professionals provided them. Overweight and obese patients were more likely to receive weight control education. Smokers were more likely to be educated about smoking control. These results indicated that health professionals in China have the tendency to provide diabetes education based on a patient’s health behaviors and health status. However, we still should notice that there was no difference in exercise education among patients with different BMIs. Given the fact that diabetes self–management involves various activities like maintaining a normal body weight, eating healthy diet, and being physical active, failure to provide adequate and comprehensive diabetes education to diabetic patients may increase the risk of adverse health outcomes. With reference to diabetes education program development, our findings suggest that tailored diabetes education covering all aspects of diabetes knowledge are highly needed and would contribute to better diabetes self–management.

This study suffered from several limitations. First, the diagnosis of diabetes is a self–reported measure. It is possible that there is a huge undiagnosed diabetic population, especially in the rural areas. We may have different results if we recruit participants based on medical records or objective measure such as HbA1c. Second, the measure of diabetes education that the CHARLS included is crude so that we are not able to explore the specific education contents. In addition, the guideline from ADA and CDS suggests that diabetes education should include information about disease process, self–monitoring blood glucose, and individualized strategies to address psychological issues [23,24]. Due to limited information collected in the CHARLS data set, we are unable to explore whether these aspects of diabetes education is associated with place of setting. We also did not include health insurance as a contextual factor in our study because of the high colinearity with place of settings. Additionally, the CHARLS data set is cross–sectional. We could only assess the factors that are associated with disparities in receipt of diabetes education due to the nature of the data available. Longitudinal studies are essential to test the causal relationships. Finally, increasing evidence suggests that eye care and dental care are important for diabetes patients; however, the data does not list any of these as a response category. While very little attention has been paid to these care for the patients, practical guidelines and clinical practice needs to be improved to incorporate these as a part of their guideline and routine practice.

## CONCLUSION

Our study found that the receipt of diabetes education was strongly associated with people’s residential locations. Aspects of diabetes education also varied by place of residence in China. Still a large amount of diabetic patients did not receive smoking control and foot care education. Gender, age, years of diabetes, and numbers of comorbidities were significant factors associated with to people receiving different aspects of diabetes education. Individuals health behaviors were also associated the aspects of diabetes education given by health professionals. However, we need to be aware that the results are based on self–reported information on the receipt of diabetes education; thus, the results may be subject to recall bias.

## IMPLICATIONS

Our study suggests that expanding outpatient care coverage and providing more tailored and comprehensive education are crucial for facilitating diabetes self–management and preventing diabetes complications for middle–aged and older adults in China. In addition, health policies should promote the strengthening of community–based non–communicable diseases health education and health promotion based on community health capacity building. In China, nurses serve as diabetes educators – a critical element in diabetes management. The shortage of nurses and uneven distribution of health care facilities require more adequate training for nurses to be able to educate diabetics self–managing their diseases. In addition, some innovative diabetes education interventions such as mobile health programs are urgently needed as a supplement of health professional diabetes education.
